# Comparative iTRAQ-based quantitative proteomic analysis of the Chinese grass shrimp (*Palaemonetes sinensis*) infected with the isopod parasite *Tachaea chinensis*

**DOI:** 10.1186/s13071-019-3675-5

**Published:** 2019-08-23

**Authors:** Yingdong Li, Xin Li, Weibin Xu, Zhibin Han, Yingying Zhao, Jing Dong, Hua Wei, Qijun Chen

**Affiliations:** College of Animal Science and Veterinary Medicine, Shenyang Agricultural University, Dongling Road 120, Shenyang, 110866 China

**Keywords:** Comparative proteomics, Isopod parasite, *Palaemonetes sinensis*, *Tachaea chinensis*, Isopod parasite

## Abstract

**Background:**

Although parasitic isopods can negatively affect the reproduction and ingestion of several commercially important crustaceans, little is known regarding the mechanisms that underlie these effects.

**Methods:**

In the present study, the iTRAQ (isobaric tags for relative and absolute quantification) approach was applied to identify differentially expressed proteins in the Chinese grass shrimp *Palaemonetes sinensis* infected with the parasitic isopod *Tachaea chinensis*.

**Results:**

On the basis of our analysis, we identified 1262 proteins from a total of 4292 peptides. There was a significant difference in the expression of 182 proteins between the control and infected groups, among which 69 were upregulated and 113 were downregulated after *T. chinensis* infection. The differentially expressed proteins revealed that parasitism may inhibit the immune response, thereby increasing host vulnerability to additional lethal infection. Furthermore, *T. chinensis* may secrete anticoagulants to inhibit hemolymph clotting. Moreover, the isopod parasite placed a heavy metabolic burden on the host, particularly with respect to glucose metabolism.

**Conclusions:**

Our study is the first to use the iTRAQ-based proteomic approach to analyze the effects of an isopod parasite on its host. The results we obtained using this approach will make a valuable contribution to understanding the molecular mechanisms underlying isopod parasitism on crustaceans.

## Background

Parasitic isopods have a negative impact on a variety of commercially important fish and crustacean hosts [1]. The rapid development of aquaculture has led to increased international interest in parasites; however, unlike for parasites such as protozoans, trematodes, copepods and acanthocephalans, there have been relatively few studies on isopod parasites. Although many researchers have focused on the phylogenetic systematics [2–4], life history [5–7] and epidemiology [1, 8] of parasitic isopods, many aspects of the molecular mechanisms underlying the negative impacts on the host, as well as host responses, remain poorly understood.

Ectoparasitic isopods feed primarily on blood (hemolymph) that oozes from wounds of the host after the integument has been perforated by the isopod’s mandibles, which can cause host stress, tissue damage, secondary infection and mortality [1, 8–10]. Although trypsin inhibitors and anticoagulants have been found in isopod parasites, the mechanism by which it evades the immune response of the host during parasitism is still unclear; Manship et al. [11] proposed that the mechanism may include hemostasis, which limits blood loss by vasoconstriction and platelet aggregation. This is similar to the mechanism used by ticks [11]. Moreover, most studies have tended to focus on isopod–fish interactions, whereas comparatively little attention has been devoted to the interactions between isopods and crustaceans. To date, numerous approaches have been applied in studying the defense system response of crustaceans during pathogen infection, including high-throughput expressed sequence tag analysis, suppression subtractive hybridization and simple gene investigation [12–14]. In a previous study, we identified a number of critical genes related to the host response after isopod parasitism [12]; however, mRNA may not provide information regarding the presence of different protein isoforms or the posttranslational modifications of proteins. Therefore, an investigation of changes in the host proteome is important, since proteins, unlike transcripts, directly reflect the host’s response [15, 16].

*Tachaea chinensis*, one of the common ectoparasites of economic shrimps, is widely distributed in China and neighboring countries [17]. *Tachaea chinensis* is approximately 0.8 cm long, mainly attaches to the ventral thoracic region of shrimps and can readily be detected by the naked eye after parasitization (Additional file 1: Figure S1). In the present study, we performed a quantitative proteomic profiling using isobaric tags for relative and absolute quantification (iTRAQ) of the Chinese grass shrimp, *Palaemonetes sinensis.* The profiling was performed for shrimps infected with a parasite and for uninfected control shrimps to determine the integrated molecular mechanisms underlying *T. chinensis* parasitization and the resultant host responses. Our data fill a major knowledge gap in research on parasitic isopods, and thereby provide an important empirical basis for disease prevention and control efforts. Additionally, our results should support further research on the molecular biology of isopods.

## Methods

### Animals

The *T. chinensis* (1.24 ± 0.13 cm) used in this study were collected from a rice field in Panjin City, Liaoning Province, China, in April 2018, and transported to the aquaculture laboratory at Shenyang Agricultural University. *Palaemonetes sinensis* (3.48 ± 0.35 g) were caught using a net cage in a lake nearby the laboratory (no isopod parasitism had previously been reported in this area). These shrimps were acclimated in two 300-l square fiberglass recirculation tanks. Each tank was linked to a circular flow system. The water temperature was maintained at 22 ± 0.5 °C and the photoperiod was set at a light:dark cycle of 12:12 h. After 2 weeks of acclimation, 20 healthy *P. sinensis* individuals (3.16 ± 0.41 cm, *n* = 20) were transferred into individual plastic tanks (15.8 cm diameter and 32.1 cm height), each of which contained 5 l of water from the acclimation tanks, and environmental conditions were the same as those used for the acclimation period. Subsequently, 10 *T. chinensis* specimens were transferred into 10 tanks separately (one per host) and formed the infected group. The shrimps in the remaining 10 tanks were used as the controls. According to the results of our previous study, most parasitized shrimp began dying after 15 days (unpublished data); therefore, after 7 days in the present study, all 10 control shrimps and all 10 infected shrimps (without parasites) were placed separately in twenty 2-ml RNAse-free tubes that were immediately frozen in liquid nitrogen for storage until protein extraction.

### Protein digestion and iTRAQ labeling

For each sample, 200 µg of protein was diluted in 200 µl of uric acid (UA) buffer (8 M urea, 150 mM Tris-HCl, pH 8.0) and transferred onto a 10-kDa ultrafiltration filter. The samples were centrifuged at 14,000× *g* for 15 min and washed with 200 µl of UA buffer. After incubation in 100 µl of 50 mM iodoacetamide in UA buffer for 30 min in the dark, the samples were centrifuged again at 14,000× *g* for 10 min. The filters were washed three times with 100 µl of UA buffer and then twice with 100 µl of dissolution buffer. Finally, 2 μg of trypsin (iTRAQ Reagents, SCIEX, Foster City, USA) was added to each filter and digested overnight at 37 °C.

iTRAQ labeling was performed using an iTRAQ Reagent-8 plex Multiplex Kit according to the manufacturer’s instructions (Applied Biosystems, Foster City, USA). The proteins in the control group were labeled with reagent 118, 119 and 120, whereas those in the infected group were labeled with reagent 121, 122 and 123.

### SCX fractionation and LC-MS/MS analysis

The iTRAQ-labeled samples were purified by strong cation exchange (SCX) chromatography using a Polysulfoethyl 4.6 × 100 mm column (5 μm, 200 Å; PolyLC Inc, Columbia, MD, USA) in an AKTA Purifier 100 (GE Healthcare, Piscataway, USA). After SCX fractionation, 33 fractions were collected and combined into 10 pools and desalted on C18 cartridges (Sigma-Aldrich, Saint Louis, USA). Each fraction was segregated using a nano HPLC Easy nLC system (Thermo Finnigan, Hemel Hempstead, UK) incorporating two Thermo Fisher Scientific (Hemel Hempstead, UK) EASY columns (2 cm × 100 μm, 5 μm-C18 for sampling and 75 μm × 100 mm, 3 μm-C18 for analysis). The flow rate was set at 250 nl/min. The peptides were eluted with a gradient of mobile phase A (0.1% formic acid in water) for 5 min, and then in a gradient starting from 0 to 35% mobile phase B (0.1% formic acid in 84% acetonitrile) for 45 min, followed by an 8 min linear gradient to 100%. Finally, the samples were maintained in 100% mobile phase B for 2 min. Each sample was subjected to mass spectrometry (MS) survey using a Q-Exactive mass spectrometer (Thermo Finnigan). Briefly, a full MS survey scan was performed for a mass range of 300 to 1800 m/z with resolution of 70,000 at m/z 200. High energy collisional dissociation (HCD) fragmentation was used for MS/MS, and the 10 most intense signals in the survey scan were fragmented. Normalized collision energy was set at 30 eV with a 0.1% underfill ratio.

### Data analysis

The raw files were analyzed by using Proteome Discoverer v.1.4 software (Thermo Fisher Scientific, Karlsruhe, BW, Germany). Identification of the proteins was conducted using the MASCOT v.2.2 search engine (Matrix Science Ltd., London, UK). The protein identification and quantitation parameters are shown in Table 1. Differential proteins were analyzed for significant down- or upregulation, which was calculated using Protein Pilot. The values of the intensities of the three reporter ions for each experimental group were averaged and then the difference was statistically assessed. The fold change was set to > 1.2 for protein upregulation and < 0.83 for protein downregulation.

**Table 1:** Upregulated proteins after parasitization

Description	Accession no.	Coverage	Proteins	AAs	MW (kDa)	Ratio (Infected/Control)	*P*-value
14-3-3 epsilon-like protein variant 1	A0A385L4D7	18.13	1	160	18.1	1.22	0.001
Acetone stress-related protein	Q5I5X1	2.76	1	254	28.9	1.48	0.002
Bestrophin homolog	A0A0P4W316	1.04	2	576	64.1	1.53	0.001
Carbohydrate sulfotransferase	A0A0P4WDX4	2.19	2	274	31.5	1.27	0.006
Chitinase	X2C079	6.51	4	261	29.5	1.73	0.041
Cuticular protein 34	A0A0B5J4U1	3.03	1	330	33.9	1.42	0.002
Cytochrome c oxidase subunit 1	B2BZU5	22.41	4	116	12.7	2.23	0.001
Cytochrome c oxidase subunit 1	A0A2S1ZYM8	6.19	1	210	22.5	1.34	0.008
Cytochrome c oxidase subunit 1	R9YY66	6.08	4	181	19.5	1.35	0.017
Cytochrome c oxidase subunit 2	A4U7M4	10.26	6	156	17.8	1.36	0.001
Cytochrome c oxidase subunit 3	A0A385JEL2	5.73	1	262	29.8	1.28	0.036
Fatty acid synthase	A0A336T938	0.45	4	2462	263.3	1.32	0.001
Ferritin	A0A2I5R2N2	10.59	1	170	19.3	1.32	0.007
Glyceraldehyde 3-phosphate dehydrogenase	A0A097KWH6	62.15	87	177	18.3	1.52	0.001
Heat-shock protein 70 kDa	A0A1B1FH77	22.14	32	524	57.3	1.31	0.005
Histone deacetylase	A0A0P4VPX5	1.45	11	1033	112.9	1.22	0.009
Histone H3	A0A1P8SG90	64.22	205	109	12.3	1.27	0.002
Hyperglycemic hormone	A0A096XHN8	26.81	1	138	15.6	2.43	0.018
Integrin beta	V9I2Y6	1.41	2	782	86.9	1.37	0.002
Male reproductive-related protein A	B8LG23	9.33	1	150	14.7	1.32	0.001
NADH-ubiquinone oxidoreductase chain 4	A0A344GDL5	2.21	1	453	51.1	1.52	0.002
Paramyosin	D7F2L7	50.00	6	68	8.0	1.32	0.031
Peritrophin	C5HYF3	7.48	1	107	12.0	1.31	0.001
RBR-type E3 ubiquitin transferase	A0A0P4WLC4	2.18	1	505	59.4	1.59	0.025
Ribosomal protein L12	G0ZJA7	8.49	3	106	11.5	1.37	0.001
Slow muscle myosin S1 heavy chain	D7F2L5	80.73	11	218	25.3	1.45	0.002
Transient receptor potential cation channel subfamily M-like protein	A0A1L3INV1	1.20	7	916	104.5	1.68	0.031
Troponin C	D7F1Q2	28.67	11	150	16.8	1.35	0.001
Tubulin alpha chain	O01942	29.27	1	451	50.3	1.56	0.032
Ubiquitin carboxyl-terminal hydrolase	A0A0P4WK80	2.19	3	228	25.3	1.31	0.001

These proteins were linked to the following databases for downstream analysis: Quick GO (Gene Ontology analysis), KOG (Clusters of orthologous groups for eukaryotic complete genomes) and KEGG (Kyoto Encyclopedia of Genes and Genomes).

### Verification of the proteome data

The proteome data were confirmed at both the transcriptional and protein levels. At the transcriptional level, the significantly expressed proteins were compared with the expression of their coding genes in our previous transcriptome data. Moreover, 55 shrimps were used to validate the proteome by using the activity of cytochrome c oxidase (CCO) and hemocyanin content: 5 for the control and 50 for the experimental group. The *T. chinensis* (0.88 ± 0.15 cm) and *P. sinensis* (3.61 ± 0.29 g) were collected in November 2018. After acclimation for 2 weeks, each of the 50 shrimps in the experimental group were artificially infected with a single isopod and randomly divided into five groups. One shrimp was collected from each group at 0 (control), 24, 48, 72, 96 and 120 h after the artificial infection; from these, 200 μl of hemolymph was extracted and divided into two aliquots. Each 100-μl subsample was centrifuged for 10 min (900× *g*, 4 °C) and the resulting supernatants were immediately used for the determination of hemocyanin content and CCO activity. We detected no significant difference among groups with regard to shrimp weight.

The hemocyanin measurements were performed spectrophotometrically. The supernatant samples were diluted with 1.9 ml of buffer (10 mmol/l CaCl_2_, 50 mmol/l Tris-HCl, pH = 8.0) and readings were taken at 334 nm (O.D._334_). The concentration of hemocyanin was calculated using the following equation: hemocyanin content (mg/ml) = 2.33 × O.D._334_ × 100. CCO activity was measured using a CCO testing kit (A090-1-1, Nanjing Jiancheng Biological Product, Nanjing, China) according to the manufacturer’s guidelines.

### Statistical analyses

Statistical analyses were performed using analysis of variance (ANOVA). Significant differences between means were determined by Duncan’s test at a significance level of *P* < 0.05. All statistical analyses were performed using SPSS 22.0 software (version 22.0; IBM, Armonk, NY, USA).

## Results

### Protein profiling

**Fig. 1:**
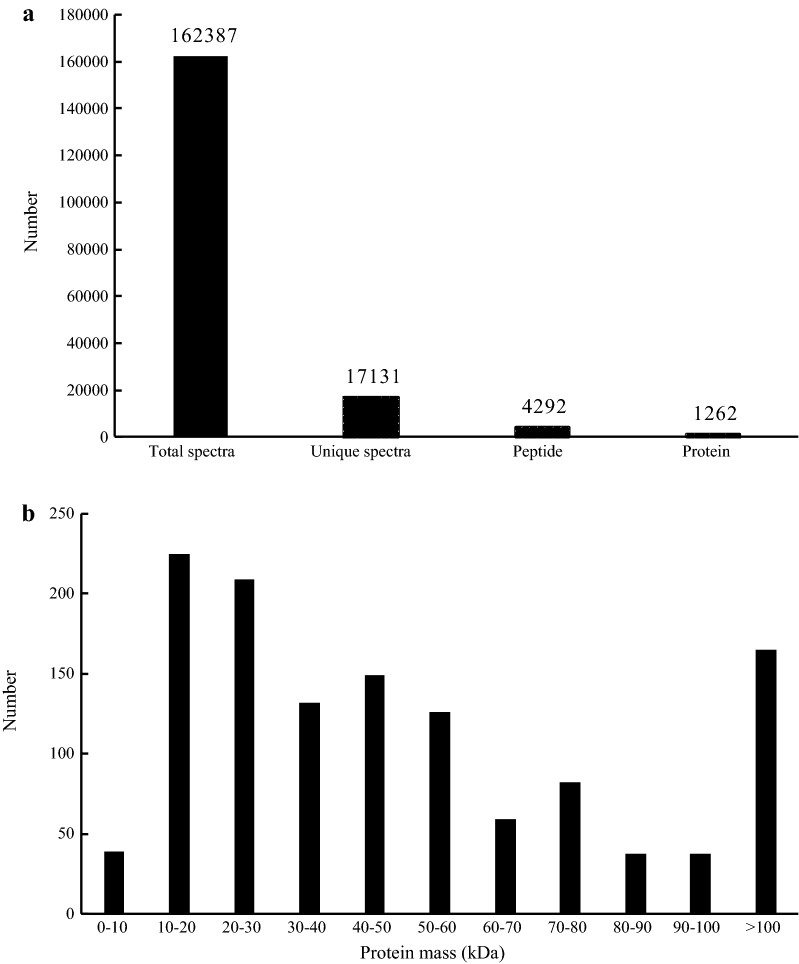
Basic information statistics. **a** Coverage of proteins by the identified peptides. **b** Distribution of identified proteins among different molecular weights

The iTRAQ analysis of the present study showed 17,131 queries in the 162,387 spectra. The protein database contained a total of 41,095 protein sequences, and the fragment and peptide mass tolerances were set to ± 20 ppm and ± 0.1 Da, respectively. Among these, a total of 1262 unique proteins were identified across the 4292 peptides (Fig. 1a). There were 225 proteins between 10 and 20 kDa, followed by 209, 132 and 149 proteins of 20–30, 30–40 and 40–50 kDa, respectively, whereas 165 proteins had a mass of over 100 kDa (Fig. 1b).

### Functional annotation and classification

**Fig. 2:**
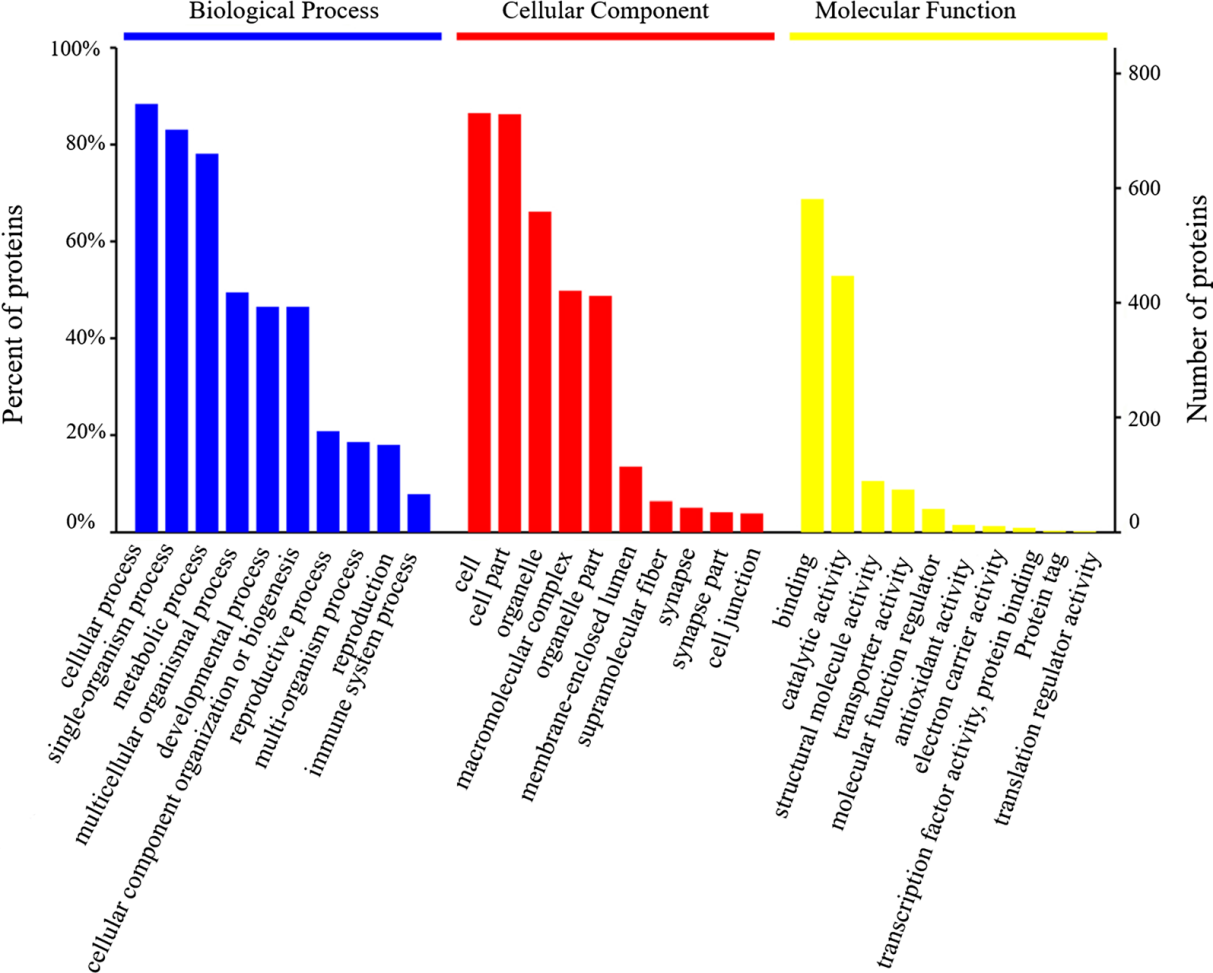
GO annotation of the identified proteins from the proteome database of the infected and uninfected shrimps. Most proteins can be divided into the three major categories: **a** biological process; **b** cellular component; **c** molecular function

**Fig. 3:**
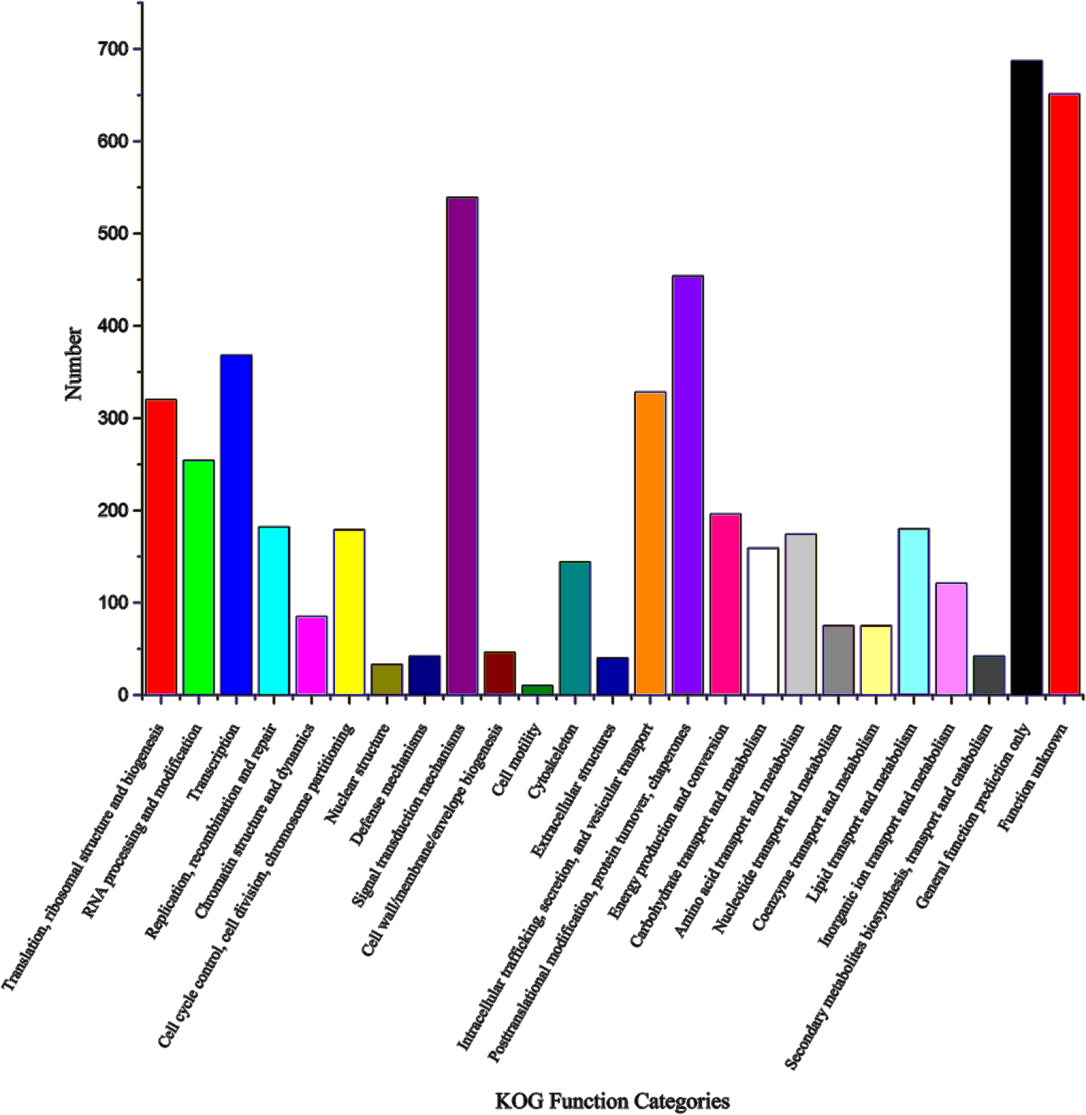
KOG annotation of the identified proteins from the proteome database of the infected and uninfected shrimps

**Fig. 4:**
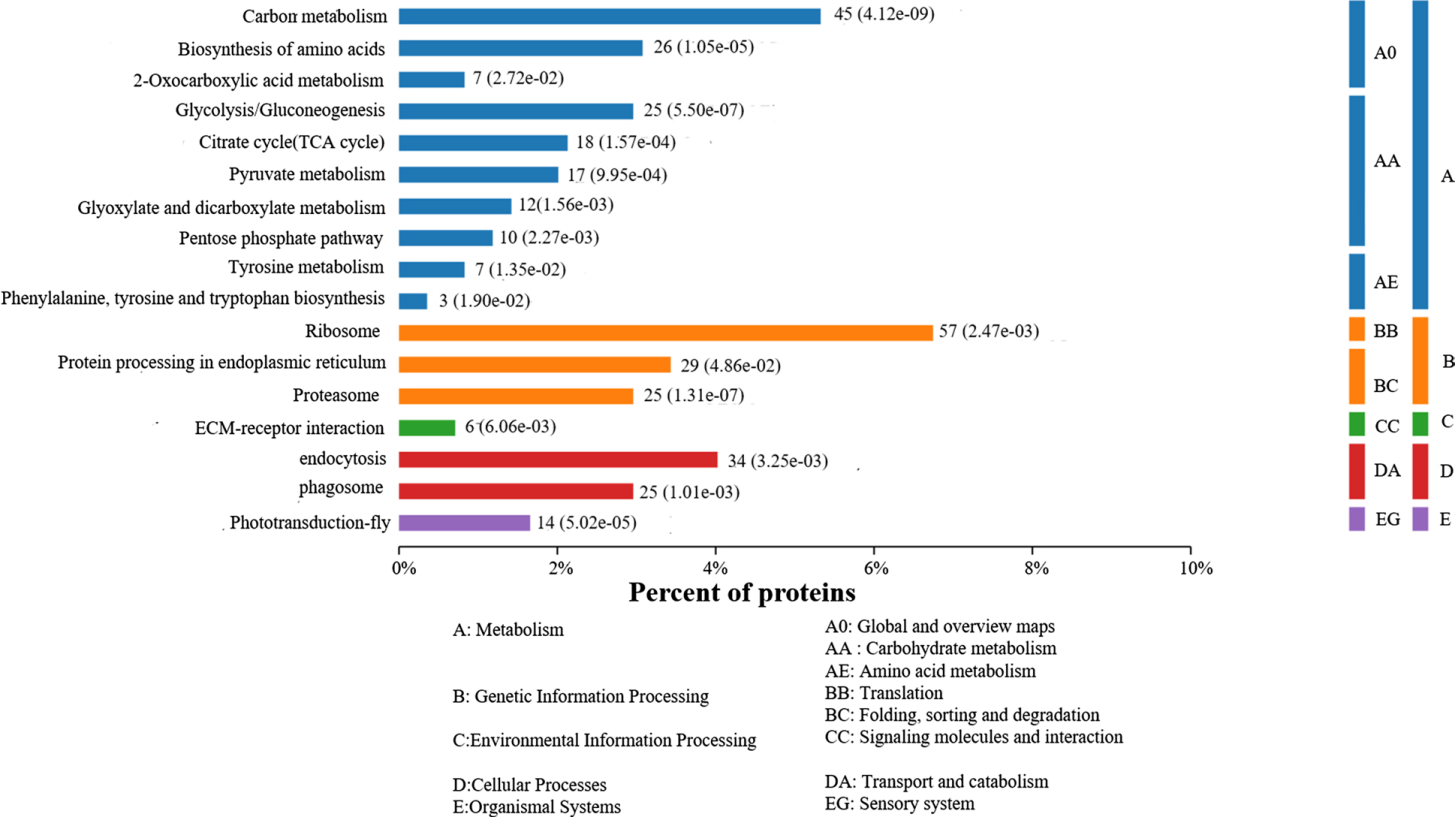
KEGG annotation of the identified proteins from the proteome database of the infected and uninfected shrimps

All 1262 unique proteins were subjected to GO, KOG and KEGG database analyses (Additional file 2: Table S1). According to the GO analysis, 824, 775 and 789 proteins were assigned to the categories “biological processes”, “molecular function” and “cellular components”, respectively (Fig. 2). The top five most frequent categories of biological processes in our study were “cellular processes” (83.96%), “single-organism processes” (82.31%), “metabolic processes” (78.93%), “multicellular organismal process” (49.15%) and “developmental process” (43.12%). To predict and classify their possible functions based on reference to orthologs from other species, all proteins were annotated using the KOG database. As shown in Fig. 3, a total of 1090 proteins were categorized into 25 groups, among which “General function prediction only” accounted for the largest group (677), followed by “signal transduction mechanisms” (541) and “posttranslational modification, protein turnover, chaperones” (452). In KEGG analysis, a total of 504 proteins were mapped against the KEGG pathway database. The most enriched pathways were as follows: carbon metabolism (45), biosynthesis of amino acids (26), glycolysis/gluconeogenesis (25), citrate cycle (18) and pyruvate metabolism (17) (Fig. 4).

### iTRAQ analysis and identification of differentially expressed proteins

Using a 1.2-fold increase or decrease in protein expression as a benchmark for a physiologically significant change, 182 differentially expressed proteins (*P* < 0.05) were identified between the control and infected groups. Among these differentially expressed proteins, 69 were upregulated and 113 were downregulated after *T. chinensis* infection (Additional file 3: Table S2). According to the GO enrichment analysis, 1691, 243 and 441 proteins were enriched for biological process, cell component and molecular function, respectively; of these 349, 62, and 129 were statistically significant (Additional file 4: Figure S2). Moreover, 78 KEGG pathways were enriched in this dataset, of which seven were statistically significant. Among the 78 KEGG pathways, 8, 8, 6, 6 and 4 mapped to the top five enriched pathways “carbon metabolism”, “phagosome”, “glycolysis/gluconeogenesis”, “biosynthesis of amino acids” and “photo transduction-fly”, respectively (Additional file 5: Figure S3).

After eliminating the uncharacterized proteins among the 182 proteins differentially expressed between the control and infected groups, we obtained 30 upregulated proteins (Table 1) and 64 downregulated proteins (Table 2). As shown in Table 1, the differentially expressed proteins related to cytoskeletal function, including slow muscle myosin S1 heavy chain, paramyosin, bestrophin homolog, cuticular protein 34, troponin C, peritrophin and tubulin alpha chain were significantly upregulated after infection. Moreover, five cytochrome c oxidase proteins and two transient receptor potential cation channel subfamily M member proteins were also identified. The highest ratio was observed for hyperglycemic hormone (2.54). Among the 64 significantly downregulated differentially expressed proteins, 30 proteins were related to immunity, notably Toll-like receptor, cathepsin L, tachylectin and annexin. The remainder included four macroglobulin- and 20 hemocyanin-related proteins (Table 2). Moreover, actin-related proteins, including beta actin and cardiac muscle actin, were significantly downregulated.

**Table 2:** Downregulated proteins after parasitization

Description	Accession no.	Coverage	Proteins	AAs	MW (kDa)	Ratio (Infected/Control)	*P*-value
Alpha-2-macroglobulin	A0A1U8VEE0	0.66	1	1519	168.7	0.58	0.008
Alpha-2-macroglobulin	A0T1M1	2.38	5	1472	163.2	0.69	0.005
Annexin	A0A0P4WDS4	4.36	8	321	35.8	0.76	0.015
AP-1 transcription factor subunit	A0A221I039	1.71	1	293	32.7	0.53	0.023
Arginine kinase	A0A088FER7	11.52	2	356	40.3	0.73	0.021
Barrier-to-autointegration factor	G0ZJ09	20.00	1	60	6.9	0.81	0.006
Beta actin	Q5EC71	72.57	4	113	12.5	0.64	0.001
Beta actin	Q8WPD5	47.34	11	376	42.0	0.79	0.001
Beta actin	Q5QEI8	23.70	1	384	43.4	0.75	0.001
Cardiac muscle actin	U3M692	64.36	4	376	41.6	0.72	0.002
Cathepsin L	D7F2M6	7.26	1	248	27.0	0.78	0.036
Chd64	A0A0A7ES46	12.61	1	119	13.1	0.71	0.017
Chitinase 1	A0A0H4M7H5	1.85	1	542	61.0	0.78	0.018
Crustin-like protein	A2TEF5	19.64	1	112	12.2	0.77	0.001
CTLDcp2	A0A0N7ELG8	3.10	1	323	36.8	0.74	0.003
Cytochrome P450 V20	H6UXP1	3.71	5	512	59.1	0.69	0.009
Cytoplasmic actin 1	A0A346QR77	48.14	5	376	41.8	0.82	0.036
Cytoplasmic type actin 3	B6EAV2	52.13	2	376	41.8	0.81	0.041
Dihydropyrimidine dehydrogenase [NADP(+)]	A0A0P4WBQ1	0.67	1	1038	112.5	0.81	0.005
Enolase	A0A2S1ZCE2	51.69	107	118	12.7	0.74	0.023
Epoxide hydrolase	A0A0H3WF09	1.30	1	460	51.3	0.76	0.041
Esterase D/formylglutathione hydrolase	D7F2N3	9.78	1	92	10.3	0.68	0.032
Glucose-6-phosphate isomerase	Q95WL9	19.35	79	155	17.4	0.78	0.038
Glutathione S-transferase	U5YDH9	10.23	2	215	24.0	0.81	0.001
Hemocyanin	Q58NQ7	48.28	1	174	20.3	0.75	0.004
Hemocyanin	D7F2N4	61.03	7	195	22.3	0.75	0.001
Hemocyanin	D7F2N5	52.94	1	51	6.0	0.53	0.006
Hemocyanin A chain	P04254	3.20	7	657	75.6	0.71	0.007
Hemocyanin alpha subunit 1	I4EC50	18.80	9	681	78.5	0.72	0.012
Hemocyanin alpha subunit 2	I4EC51	6.75	9	681	78.7	0.63	0.019
Hemocyanin beta subunit 1	I4EC48	11.60	14	664	76.3	0.54	0.027
Hemocyanin C chain	P80096	4.24	4	661	75.8	0.54	0.029
Hemocyanin	A0A0A0PM26	40.70	10	688	79.1	0.71	0.017
Hemocyanin	F5CEX2	46.46	10	663	76.6	0.71	0.008
Hemocyanin	Q8MUH8	2.73	12	660	75.3	0.63	0.003
Hemocyanin	A0A2P1CYB2	47.27	7	677	78.4	0.71	0.001
Hemocyanin	G9DE16	34.69	9	663	76.5	0.75	0.015
Hemocyanin subunit 1	M4IQR3	14.52	8	675	78.0	0.69	0.019
Hemocyanin subunit 1-like protein	A0A342CJ38	19.58	8	674	77.6	0.72	0.035
Hemocyanin subunit 2	Q95P18	6.73	9	684	78.5	0.70	0.001
Hemocyanin subunit 2	A0A142BZ28	10.83	19	683	78.1	0.49	0.018
Hemocyanin-like protein	A0A342CJ41	34.92	10	567	65.2	0.82	0.001
Hemocyanin-like protein	A0A342CJ42	32.98	9	567	65.2	0.67	0.001
Hemocyanin-like protein	A0A342CJ43	16.45	1	152	17.1	0.68	0.001
Histone H3	A0A1I9S0T4	70.64	385	109	12.3	0.71	0.001
Importin-5	A0A286RXS5	2.06	2	679	76.1	0.72	0.019
Macroglobulin	A0A0B4KIG1	1.77	5	1470	162.9	0.68	0.035
NADH-ubiquinone oxidoreductase chain 5	A0A343XYJ5	5.24	1	572	62.9	0.72	0.001
Nicotinic acetylcholine receptor subunit alpha 11	R4JS65	3.28	1	427	48.1	0.81	0.003
Origin recognition complex subunit 1	A0A0P4W126	1.69	1	949	106.7	0.66	0.001
Pescadillo homolog	A0A0P4WF39	0.93	3	643	75.8	0.82	0.015
Phosphoenol pyruvate carboxykinase	F6JTP5	9.55	1	199	21.6	0.81	0.018
Putative clotting protein	A0A0U1W4T3	2.45	1	1712	192.4	0.71	0.001
Ribosomal protein L7	D7F1Q3	5.56	1	126	14.7	0.50	0.003
Ribosomal protein rpl13	F8QXL7	12.28	4	57	6.5	0.50	0.009
Sarcoplasmic calcium-binding protein	I2DDG2	45.60	3	193	21.9	0.53	0.036
Sodium-potassium ATPase alpha subunit	R9YZ41	52.58	64	194	21.8	0.65	0.041
Sodium-potassium ATPase	I6P4H6	63.73	113	204	22.8	0.80	0.001
Sodium-potassium ATPase alpha subunit	R4JXJ7	53.62	45	207	23.2	0.53	0.001
Sodium-potassium ATPase alpha subunit	A0A075DXD5	42.26	28	168	19.0	0.83	0.003
Tachylectin	M1FAC3	3.29	1	243	27.1	0.81	0.001
Toll-like receptor	A0A0K1RL99	1.92	1	1196	136.3	0.71	0.009
Transaldolase	A0A0P4WGI7	5.14	2	331	36.8	0.71	0.008
Vacuolar ATP synthase subunit d 1-like protein	D2DSL1	8.22	2	146	17.1	0.77	0.043

### Verification of the protein data

**Fig. 5:**
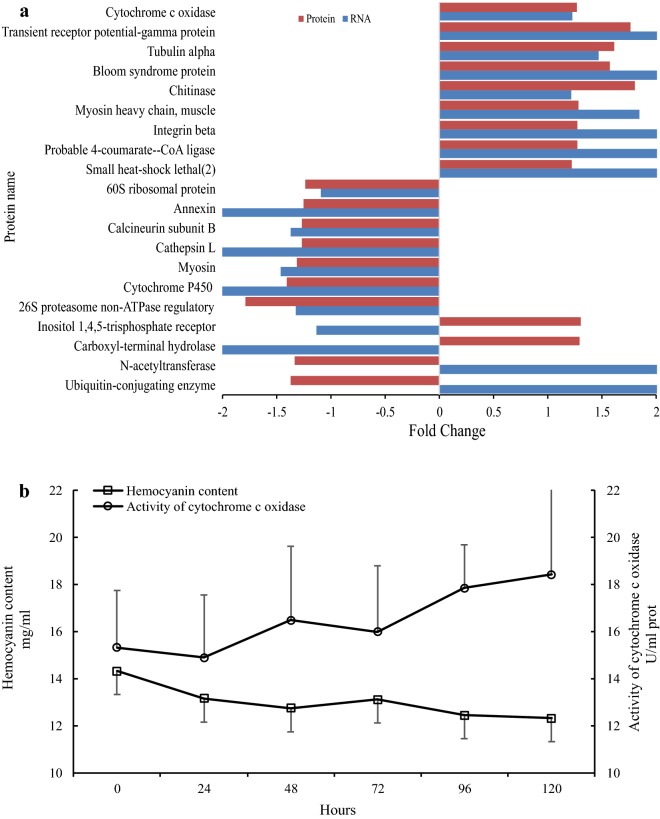
Verification of the protein data. **a** Transcriptional and proteome expression patterns of identical proteins/genes. **b** The hemocyanin content (Hc) and cytochrome *c* oxidase (CCO) activity verification

According to association analysis of the proteome and transcriptome data, 20 of the 150 differentially expressed proteins (13.33%) shared the same annotation with transcripts. Among these 20 proteins, 16 (80%) showed expression trends similar to the transcriptome expression trends in the control and infected group, including nine upregulated and seven downregulated proteins/genes (Fig. 5a).

The results of our analysis of hemocyanin content and CCO activity over a 120 h period after infection are shown in Fig. 5b. After parasitization by *T. chinensis*, the hemocyanin content of shrimps significantly decreased by 1.17 ± 0.31 (*F*_(4, 20)_ = 21.21, *P* = 0.002); 1.57 ± 0.42 (*F*_(4, 20)_ = 40.97, *P* < 0.001); 1.21 ± 0.27 (*F*_(4, 20)_ = 20.54, *P* = 0.002); 1.87 ± 0.48 (*F*_(4, 20)_ = 40.71, *P* < 0.001); and 2.01 ± 0.55 (*F*_(4, 20)_ = 72.41, *P* < 0.001) mg/ml at 24, 48, 72, 96 and 120 h after isopod parasitism, respectively. Compared with the control group (0 h), the activity of CCO significantly increased by 7.57 ± 0.88% (*F*_(4, 20)_ = 84.98, *P* < 0.001); 4.31 ± 0.46% (*F*_(4, 20)_ = 16.99, *P* = 0.003); 16.45 ± 2.54% (*F*_(4, 20)_ = 189.89, *P* < 0.001); and 20.17 ± 3.26% (*F*_(4, 20)_ = 169.89, *P* < 0.001) in shrimps parasitized for 48, 72, 96 and 120 h, respectively.

## Discussion

Investigation of the response to different pathogens represents an important research goal for elucidating immune and related components in the defense system of crustaceans [18–20]. In recent years, the isopod parasite *T. chinensis* has resulted in heavy economic losses of commercially farmed crustacea, particularly in *Penaeus vannamei*, *Macrobrachium nipponense*, *Exopalaemon carinicauda* and *Palaemonetes sinensis* [21–23]. Therefore, understanding the pathogenic mechanism of this parasitic disease has become crucial for achieving sustainable crustacean production. In the present study, using the iTRAQ-based quantitative proteomic approach, we aimed to gain a better understanding of the molecular mechanisms underlying the effects of this isopod parasite and shrimp proteome changes to the parasite.

Hemocyanin has been studied in several host-pathogen interactions, which have indicated that hemocyanin also plays an important role in the immune system of shrimps [14, 24–26] and has been found to be significantly upregulated during viral and bacterial infection [27–30]. However, patterns in the variation of hemocyanin in parasitized and healthy shrimps remain unclear. In the present study, we identified 20 hemocyanin-related proteins (31.25%) among differentially expressed proteins that were downregulated in response to parasitism. Moreover, we found that the hemocyanin content decreased concomitant with an increase in the duration of parasitism. These results are similar to those obtained for fish hosts, in which hemoglobin concentrations also decreased in response to isopod parasitization [31]. These findings indicate that *T. chinensis* could use its mandible to feed, suck the hemolymph and promote hematological changes in *P. sinensis*. Therefore, similar to other blood-feeding parasites, *T. chinensis* may also be implicated in the transmission of blood-dwelling diseases between shrimps, which we intend to examine in our future research.

Most invertebrates need to rapidly prevent the loss of blood or equivalent fluids through inflicted injuries [32]. In shrimps, the hemolymph clotting system, which comprises transglutaminase and clotting proteins, plays an important role in the innate immune response and prevention of blood loss during injury and wound healing [33]. However, in the present study, we found that a putative clotting protein, cytoplasmic type actin 3 and three macroglobulin proteins were significantly downregulated in parasitized shrimps and blood coagulation. Moreover, there was a 0.87-fold downregulation of transglutaminase, which catalyzes the gelation of the plasma (Additional file 2: Table S1). It is well known that blood-feeding parasites, such as ticks, mosquitoes, fleas and leeches need to evade the clotting system of their vertebrate hosts and maintain blood flow during feeding [34, 35]. Many researchers consider that isopod parasites may inject anticoagulants or other compounds directly into the blood to obtain their “blood meal” [11, 31]. In order to suck the hemolymph, *T. chinensis* may secrete various anticoagulants, which could work cooperatively and prevent blood clotting by downregulating the expression of clotting-related proteins at the site of injury.

Moreover, we found that five actin proteins and a Toll-like receptor protein were significantly downregulated. In contrast, these proteins have been found to be upregulated during viral and bacterial infection [36]. Crustaceans infected with viruses or bacteria exhibit a significant change in the signaling pathways related to the immune response, including MAPK, Toll-like receptor, PI3K-Akt and Jak-STAT pathways [34–37]; however, in our previous study, we detected no significant changes in these pathways during *T. chinensis* parasitization [12]. Parasite survival largely depends on circumventing the host’s immune system. Therefore, to ensure survival until transmission to the next host, parasites such as *T. chinensis* may have to balance between evading the host immune response, similar to acanthocephalans parasitizing gammarids [37, 38], and increasing host vulnerability to additional lethal infection.

According to the KEGG analysis performed in the present study, two pathways involved in glucose metabolism, namely carbon metabolism and glycolysis/gluconeogenesis, were among the top five statistically significant different pathways. Moreover, proteins associated with these two glycometabolism-related pathways, including crustacean hyperglycemic hormone (CHH), glyceraldehyde-3-phosphate dehydrogenase (GAPDH) and carbohydrate sulfotransferase (CST), were significantly upregulated. These results are similar to those obtained from our corresponding transcriptome analysis [12]. Carbohydrate dynamics and CHH concentrations were also found to be upregulated during infection with a dinoflagellate parasite in *Nephrops norvegicus* [39]. However, the upregulated levels of CHH, GAPDH, and CST were caused either directly by parasitic disruption of the shrimp’s endocrine system or indirectly by interfering with the positive and negative feedback loops.

## Conclusions

To the best of our knowledge, this is the first study to investigate the response of shrimps to an isopod parasite using an iTRAQ-based proteome method. The differentially expressed proteins related to hemocyanin content and the hemolymph coagulation system were identified as downregulated after isopod infection. This may indicate that, similar to blood sucking parasites, *T. chinensis* could inhibit the hemolymph clotting system of *P. sinensis* during parasitization. Moreover, isopod parasites place a heavy metabolic burden on their hosts, particularly with regards to glucose metabolism. Overall, our study provides valuable empirical data that will support future molecular research on isopod parasitization of crustaceans.

## Supplementary information

**Additional file 1: Figure S1.** Picture of *T. chinensis* attached to the ventral thoracic region of *P. sinensis.*

**Additional file 2: Table S1.** Total proteins compared to GO, KOG, and KEGG databases.

**Additional file 3: Table S2.** Lists of differentially expressed proteins.

**Additional file 4: Figure S2.** GO enrichment analysis.

**Additional file 5: Figure S3.** KEGG enrichment analysis.

## Data Availability

The datasets supporting the conclusions of this article are included within the article and its additional files. The mass spectrometry proteomics data have been deposited to the ProteomeXchange Consortium *via* the PRIDE partner repository with the dataset identifier PXD014542. All analyzed data are available from the corresponding author upon reasonable request.

## Abbreviations

iTRAQ: isobaric tags for relative and absolute quantification
GO: gene ontology
KOG: clusters of orthologous groups for eukaryotic complete genomes
KEGG: Kyoto encyclopedia of genes and genomes
CHH: hyperglycemic hormone
GAPDH: glyceraldehyde-3-phosphate dehydrogenase
CST: carbohydrate sulfotransferase
