# Supplementary figures and images for: Comparative iTRAQ-based quantitative proteomic analysis of the Chinese grass shrimp (Palaemonetes sinensis) infected with the isopod parasite Tachaea chinensis
Source: Parasit Vectors. 2019 Aug 23;12:415. doi: 10.1186/s13071-019-3675-5 (PMC6708196; doi:10.1186/s13071-019-3675-5)

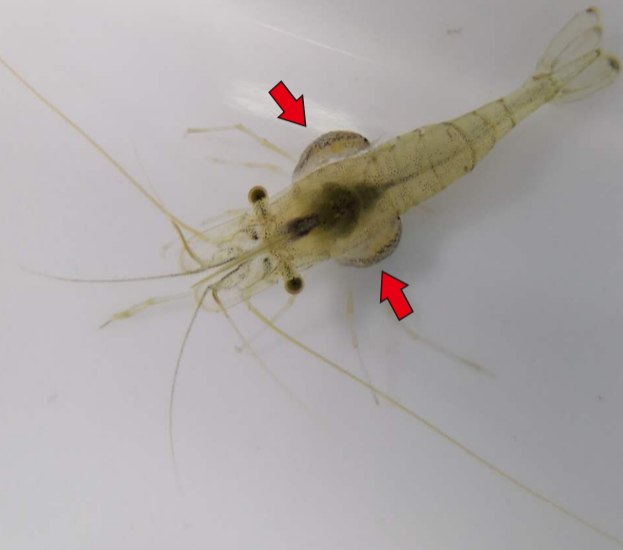

Supplement: Supplementary file 1 — Additional file 1: Figure S1. Picture of T. chinensis attached to the ventral thoracic region of P. sinensis. [file 13071_2019_3675_MOESM1_ESM.pdf]

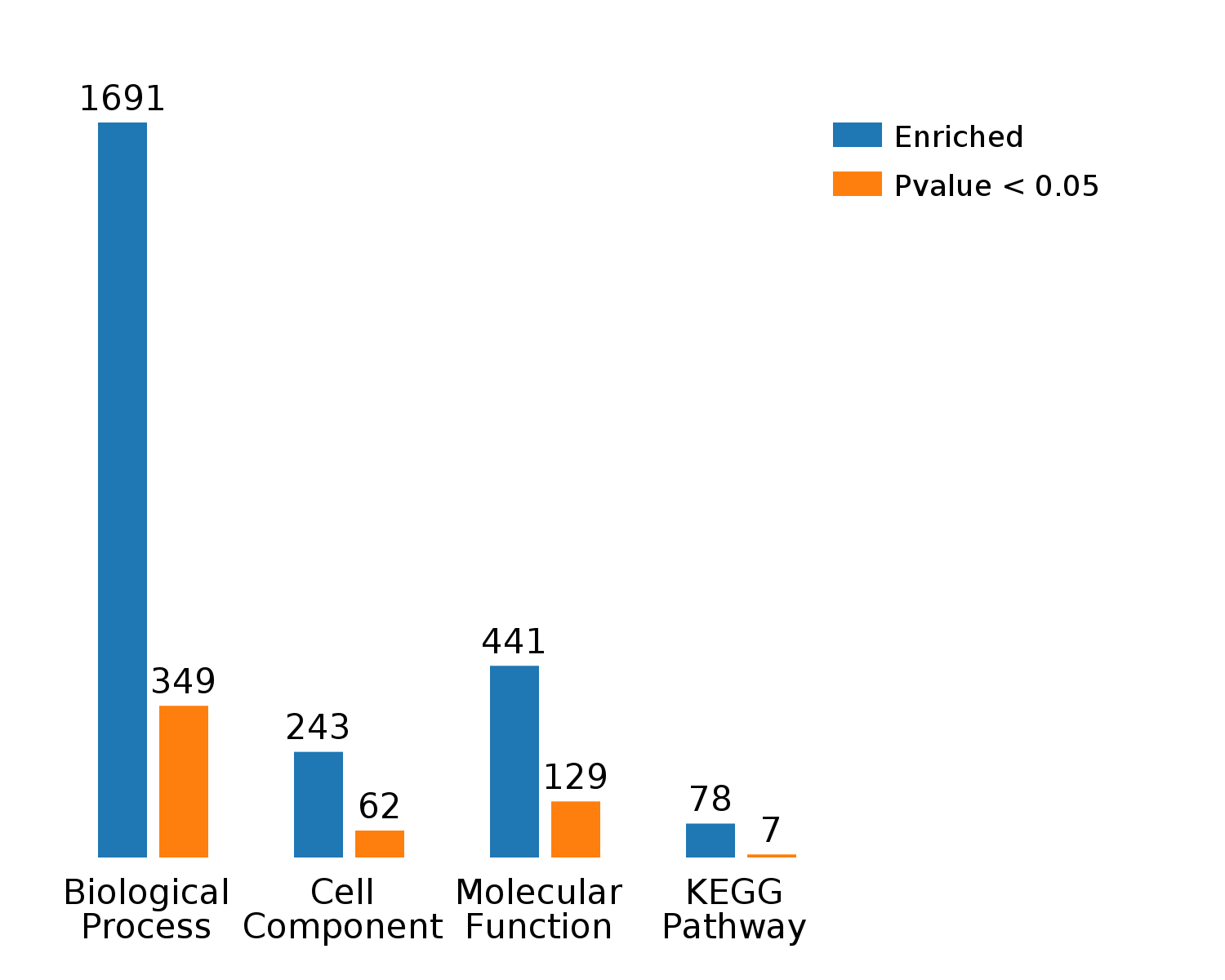

Supplement: Supplementary file 4 — Additional file 4: Figure S2. GO enrichment analysis. [file 13071_2019_3675_MOESM4_ESM.png]

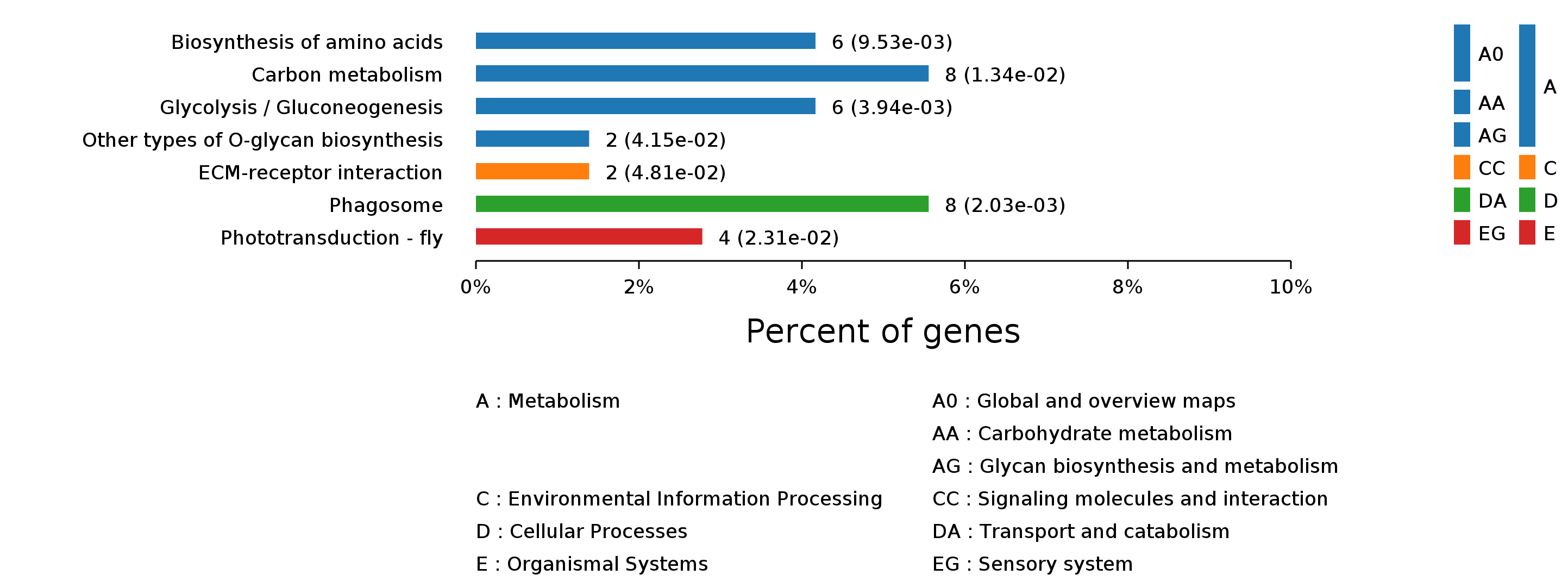

Supplement: Supplementary file 5 — Additional file 5: Figure S3. KEGG enrichment analysis. [file 13071_2019_3675_MOESM5_ESM.png]
